# Supplementary material for: Evaluating deep learning based structure prediction methods on antibody–antigen complexes
Source: Bioinformatics. 2026 Mar 21;42(4):btag136. doi: 10.1093/bioinformatics/btag136 (PMC13061134; doi:10.1093/bioinformatics/btag136)
Supplement: btag136_Supplementary_Data [file btag136_supplementary_data.pdf]

Supplementary Material

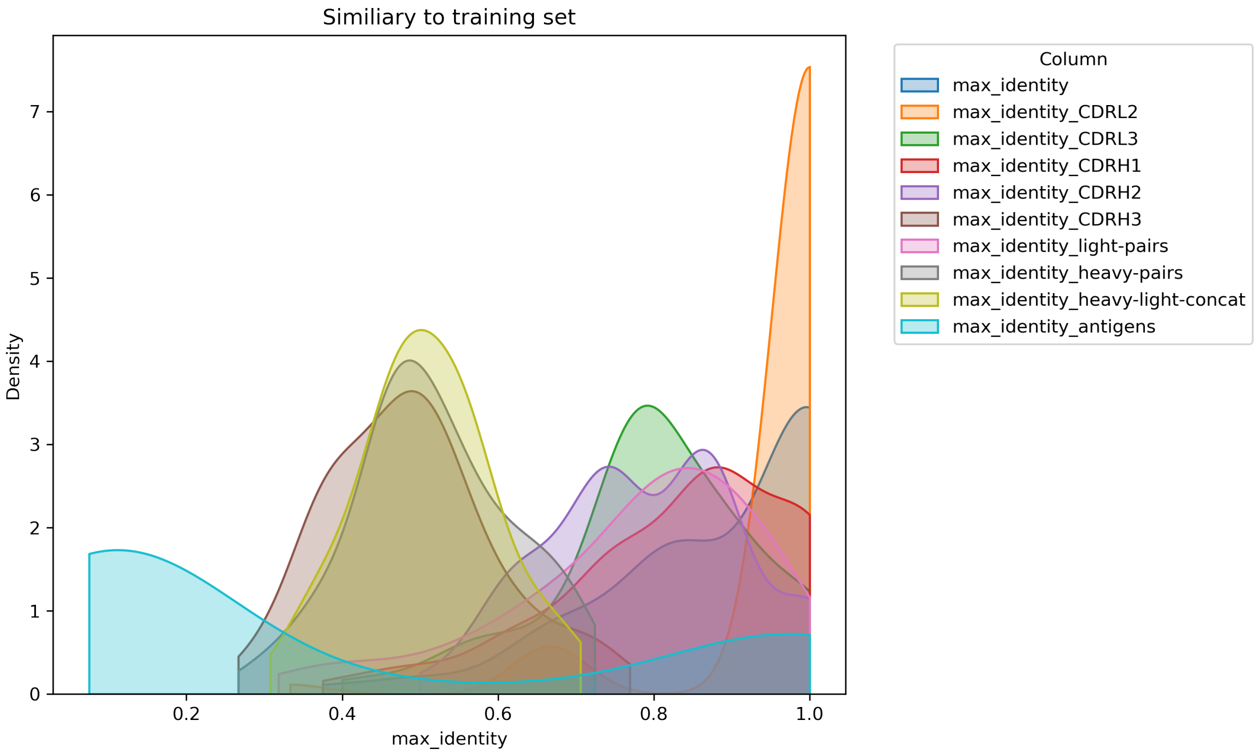

Fig. S1. Distribution of maximum sequence identity to training set for individual loops in the antibodies, concatenation of all loops in heavy/light chains or both, as well as the antigen. For all individual loops, there are almost identical sequences in the training set. In contrast, for the concatenated loops, the identity is about 50%, and for the antigens, there is a bimodal distribution with a fraction of almost identical antigens in the dataset. At the same time, a majority has less than 30% sequence identity.

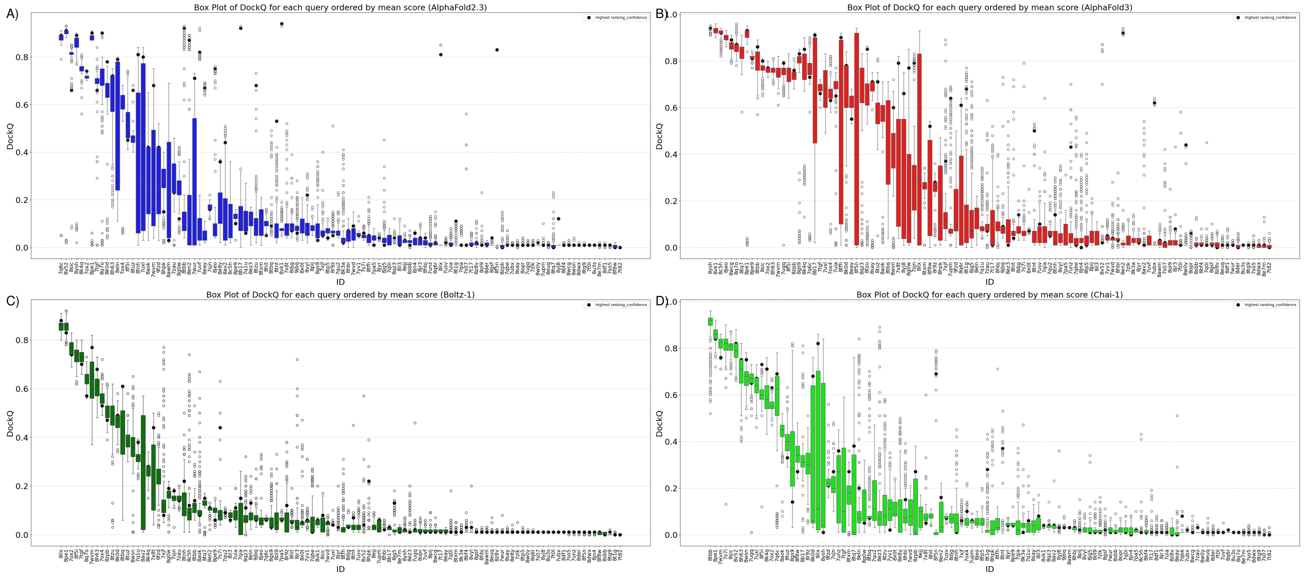

Fig. S2. Each figure shows a box plot for the DockQ score for each target ID, sorted by mean DockQ score for that target ID and that method. The highest-ranked model is highlighted with a thick dot. A) AlphaFold2.3, B) AlphaFold3, C) Boltz-1, and D) Chai-1.

.

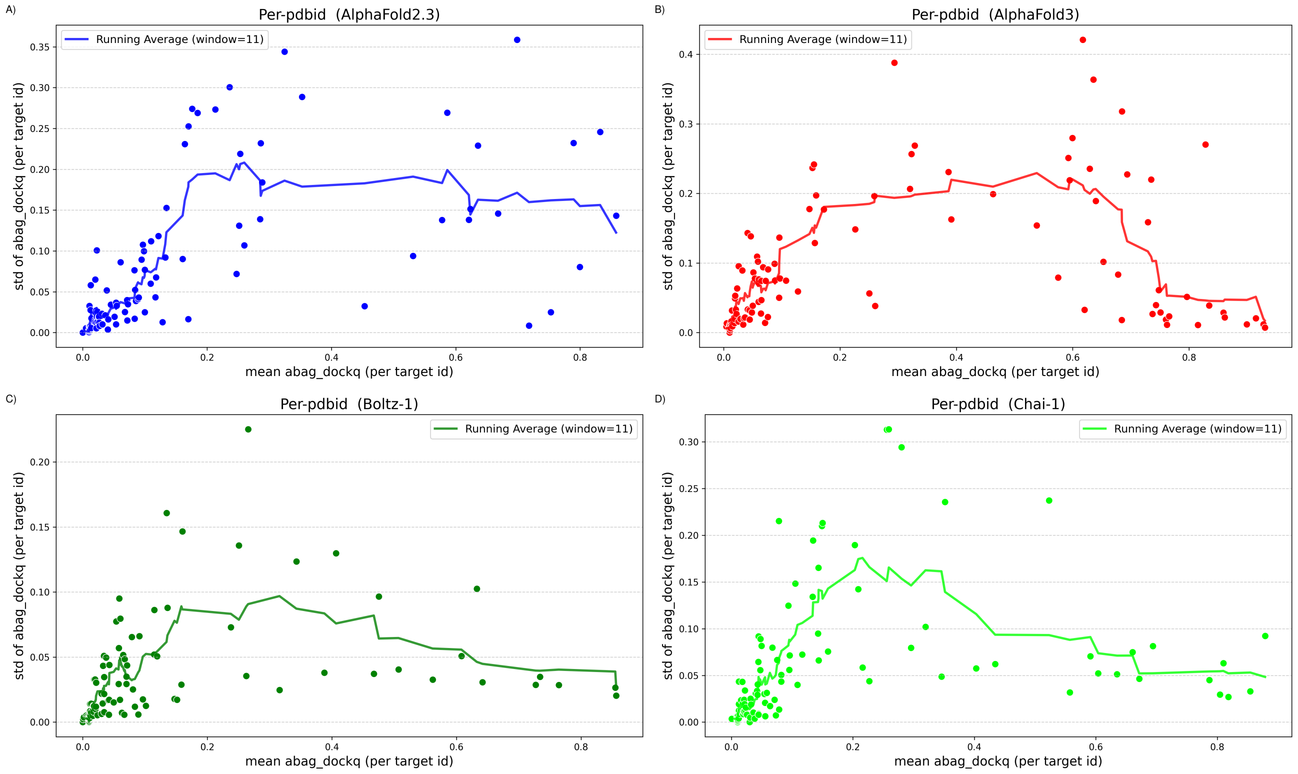

Fig. S3. Each figure shows a scatter plot for the mean vs Standard Deviation of the DockQ scores for each target. A) AlphaFold2.3, B) AlphaFold3, C) Boltz-1, and D) Chai-1. The running average (over 11 sample) show a trend that the variation is largest for "intermediately" easy targets.

.

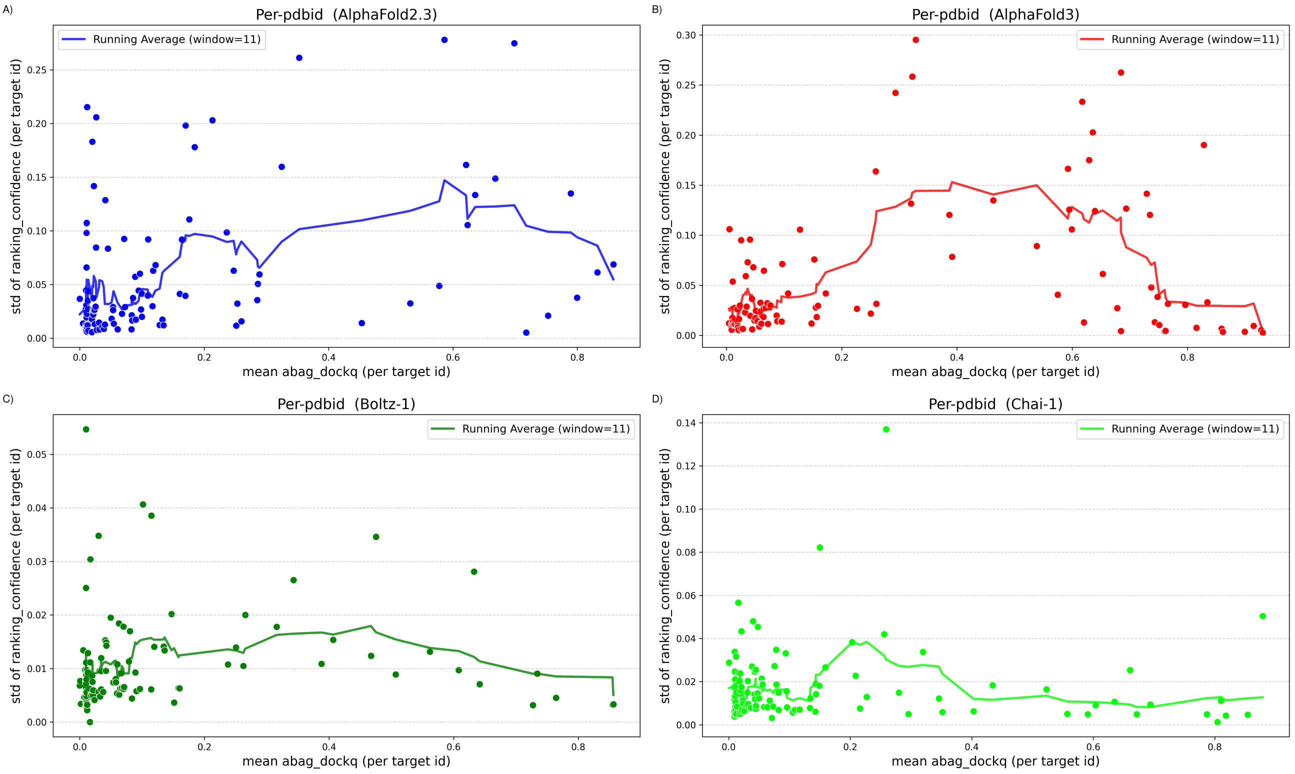

Fig. S4. Each figure shows a scatter plot for Standard Deviation of the ranking confidence scores vs the mean DockqQfor each target. A) AlphaFold2.3, B) AlphaFold3, C) Boltz-1, and D) Chai-1. The running average (over 11 sample) shows a minor trend that the variation is largest for ”intermediately” easy targets.

.

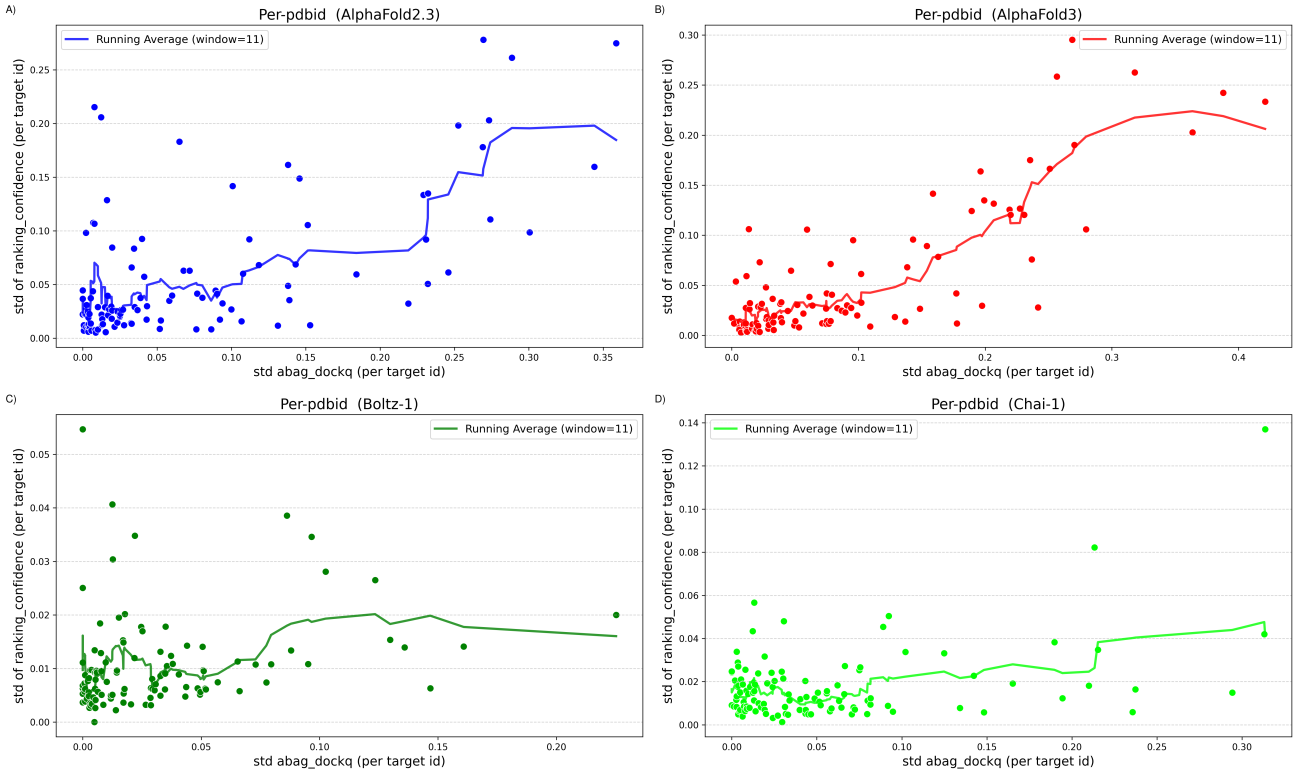

Fig. S5. Each figure shows a scatter plot for the Standard Deviation of the ranking confidence scores versus the standard deviation of the DockQ for each target. A) AlphaFold2.3, B) AlphaFold3, C) Boltz-1, and D) Chai-1. The running average (over 11 samples) shows a minor trend that the variation is largest for ”intermediately” easy targets.

.

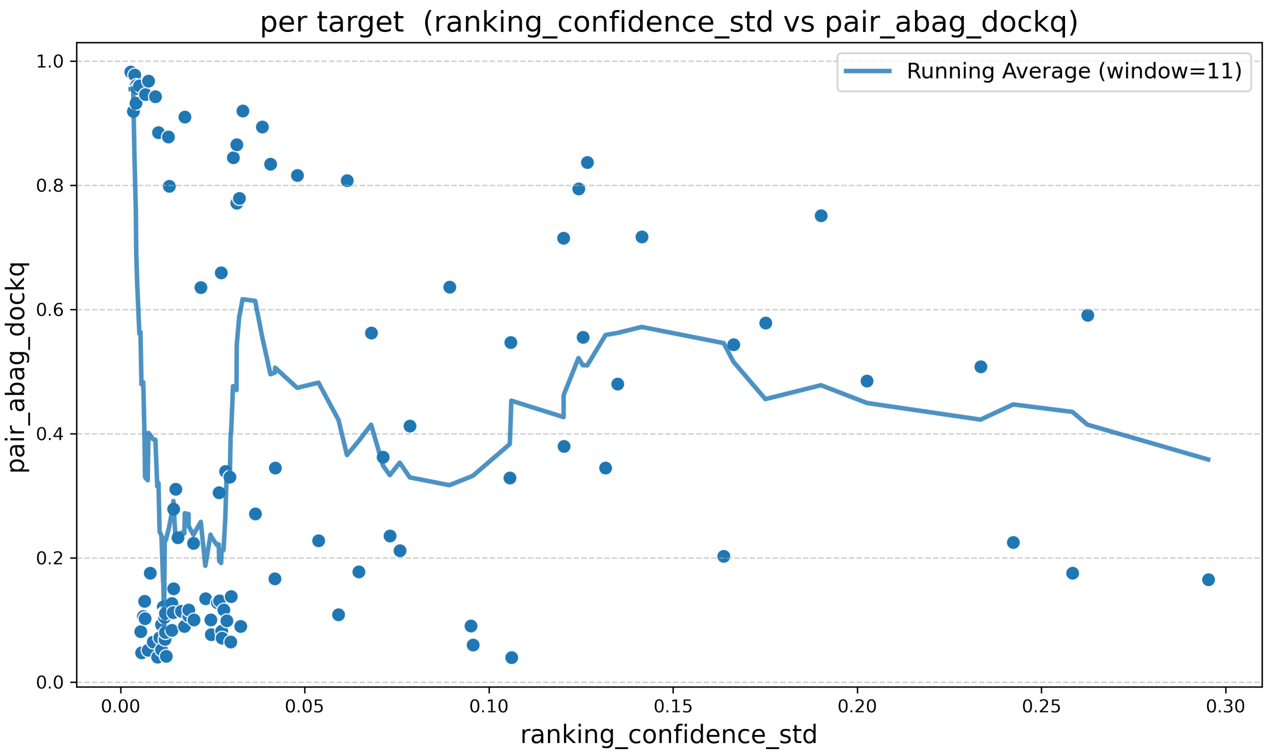

Fig. S6. Variation of ranking confidence plotted against variation of the structures, as measured by average DockQ similarity between the generated models.

.

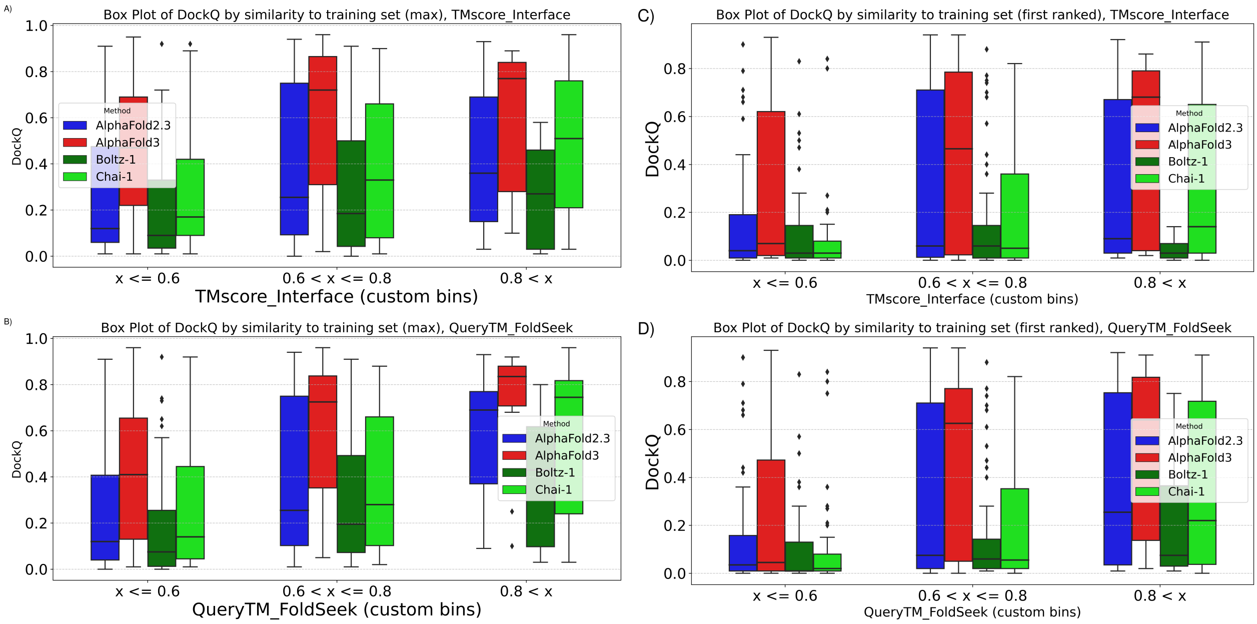

Fig. S7. Prediction performance vs. similarity to the training data for AlphaFold2.3 and AlphaFold2 using the FoldSeek *query TMscore* (A,B) or *Interface TM-score (C,D)*. Plotted for the max score in A and C and for the first-ranked scores in B and D.

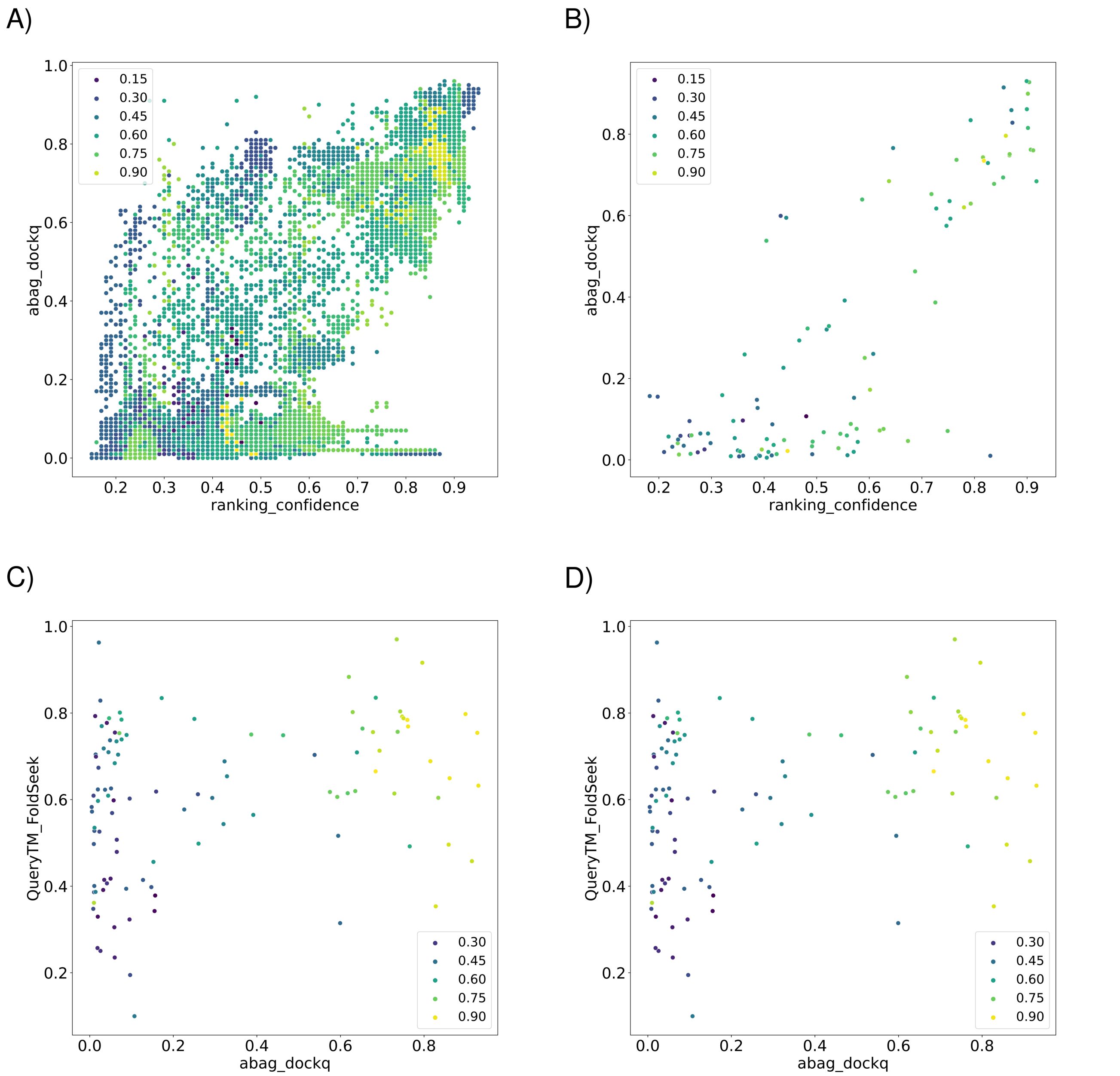

Fig. S8. A) Ranking confidence vs DockQ for all models, colouring is by maximum similarity to the training set (TM-score). B-D) The same data plotted for the mean for each target, using different projections.

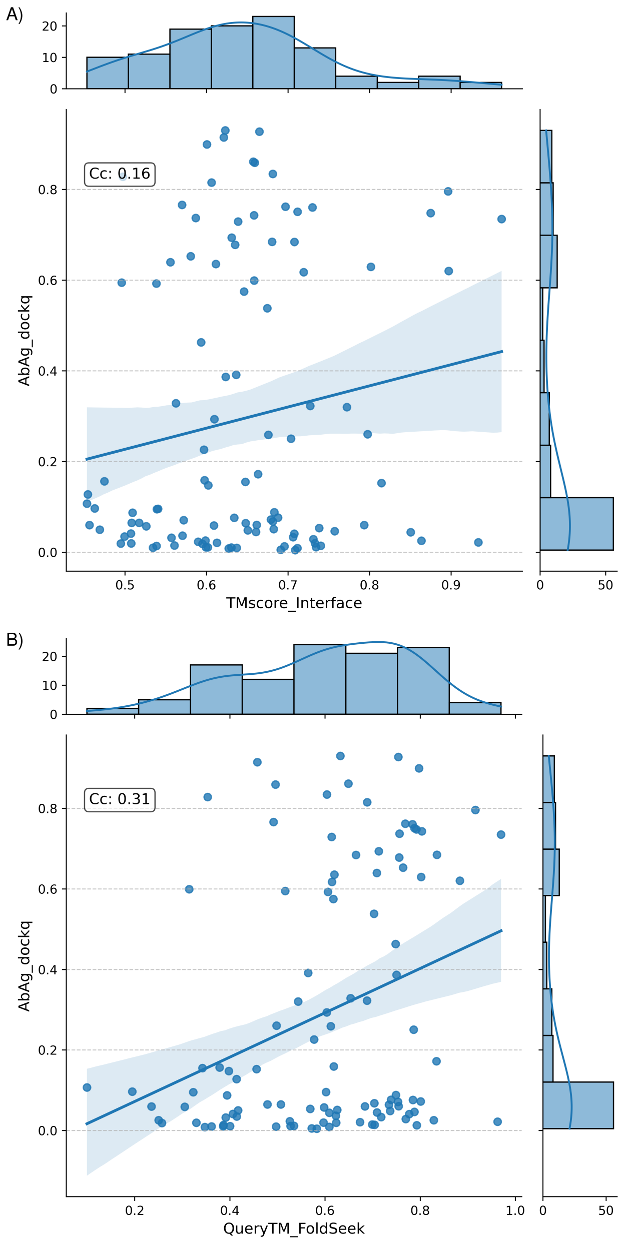

Fig. S9. Scatter plot of mean DockQ scores vs similarity to training set using interface TM-score similarity (A) or Query TMscore similarity (B) for AlphaFold3.

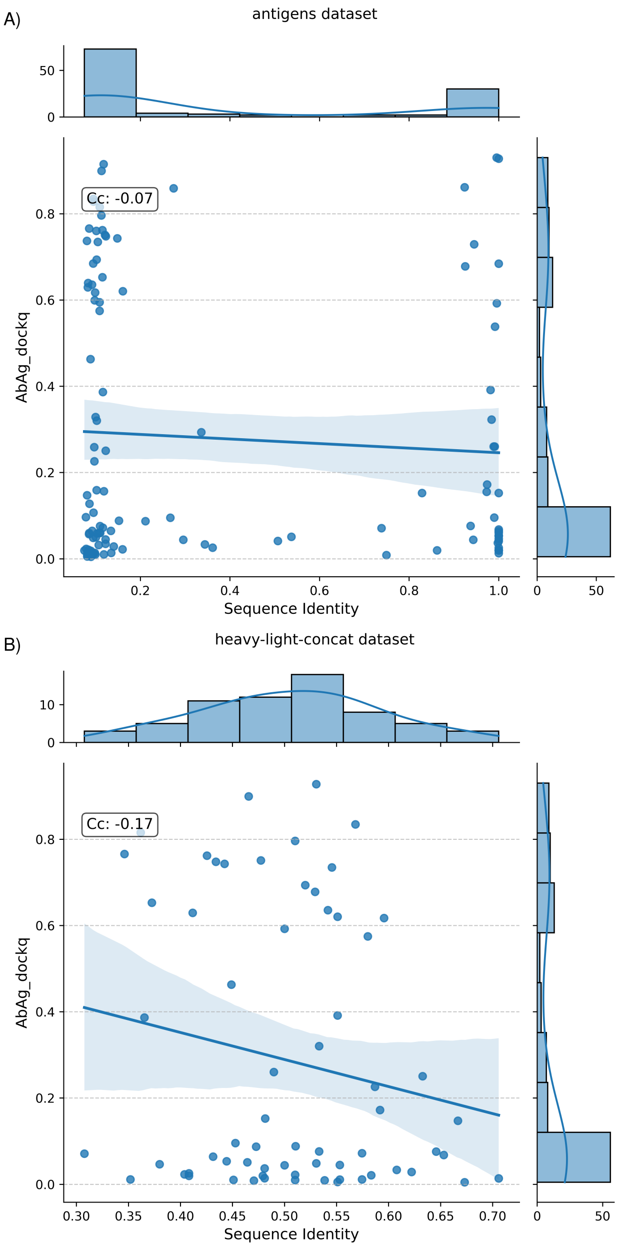

Fig. S10. Scatter plot of mean AbAg-DockQ scores vs maximum sequence identity of (a) antigens and (B) concatenated CDR loops to the training set, for AlphaFold3.

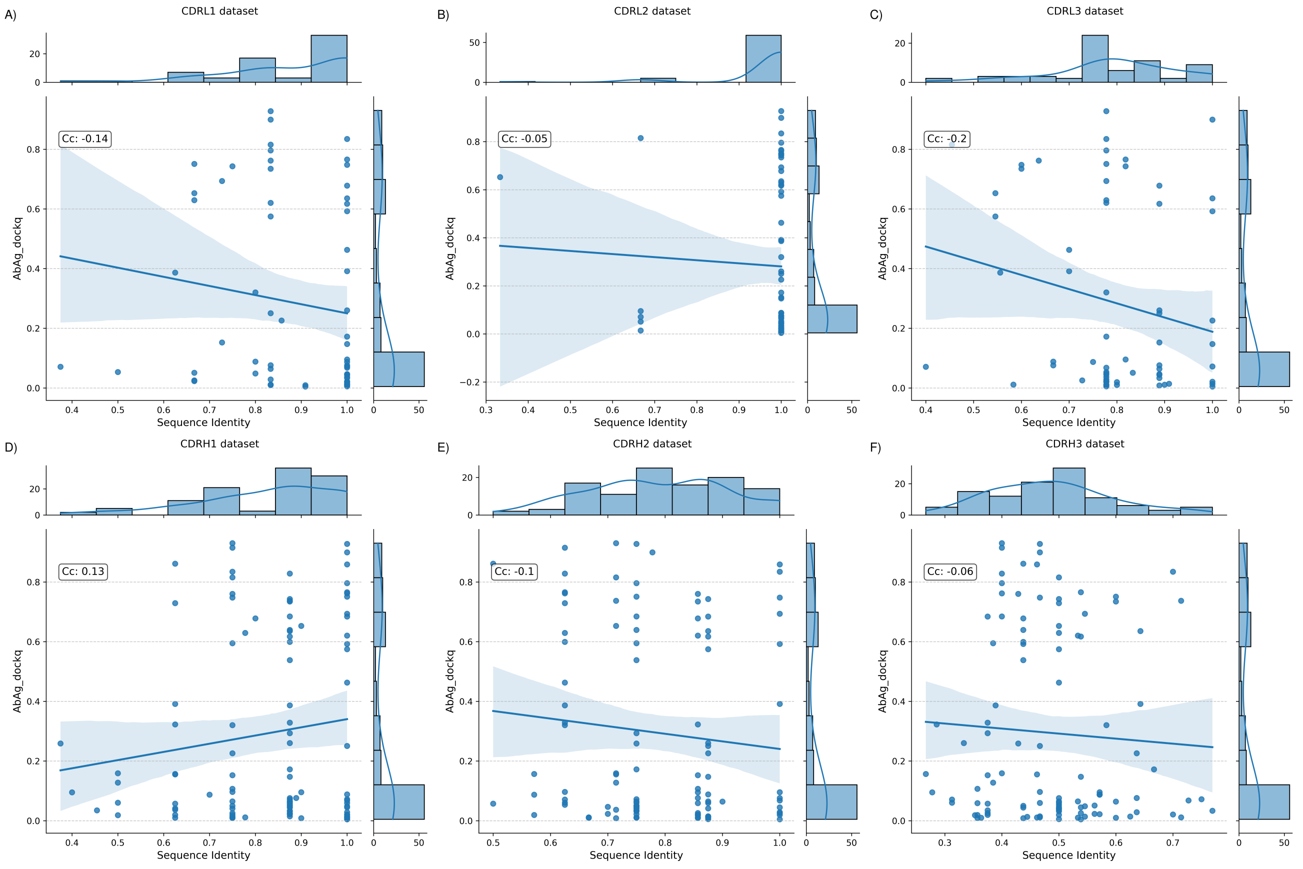

Fig. S11. Scatter plot of mean AbAg-DockQ scores vs maximum sequence identity for individual loops to the training set, for AlphaFold3.

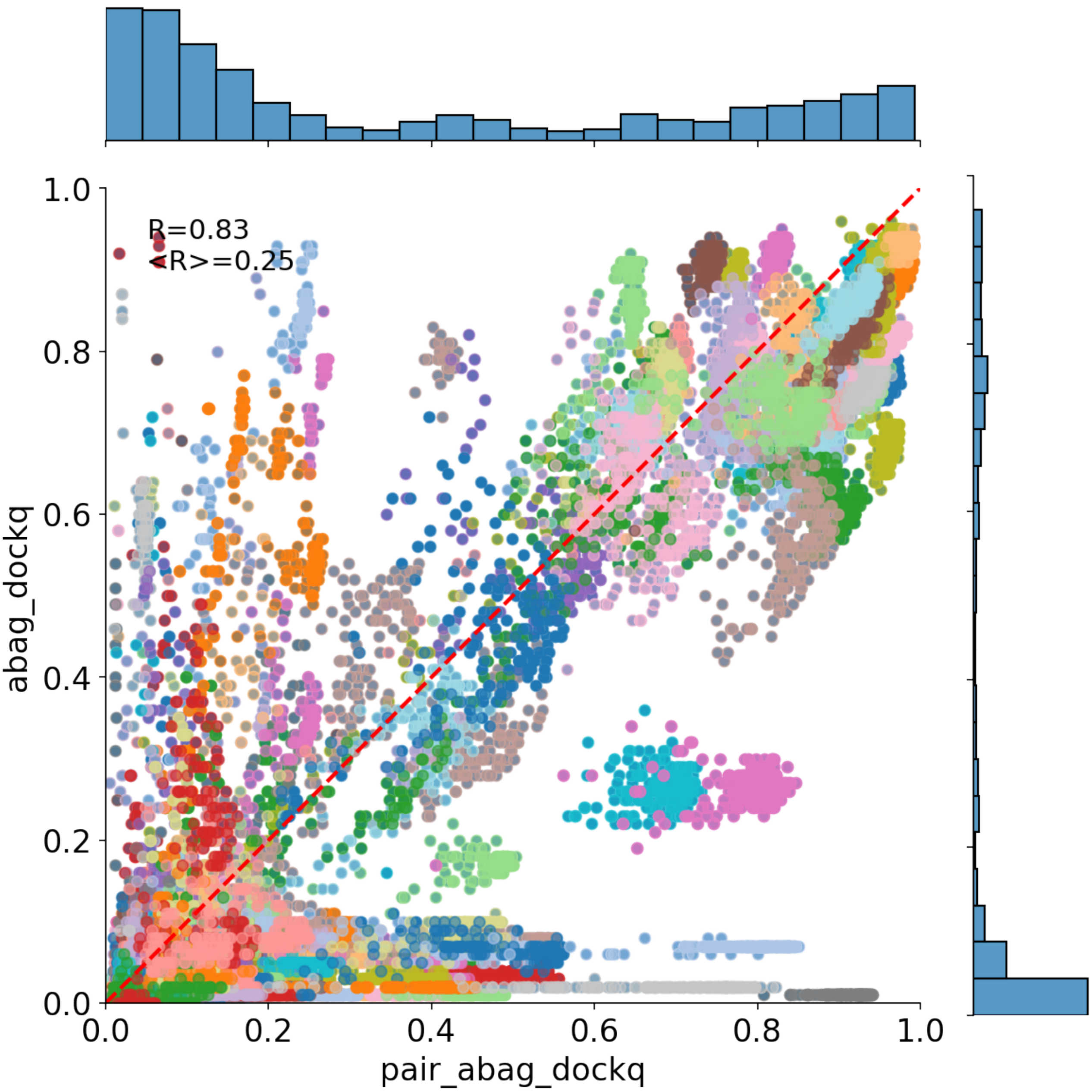

Fig. S12. Scatterplot of paired DockQ vs DockQ for all models. Each target is colored separately. The correlation overall is 0.85, while the correlation per target is 0.19.

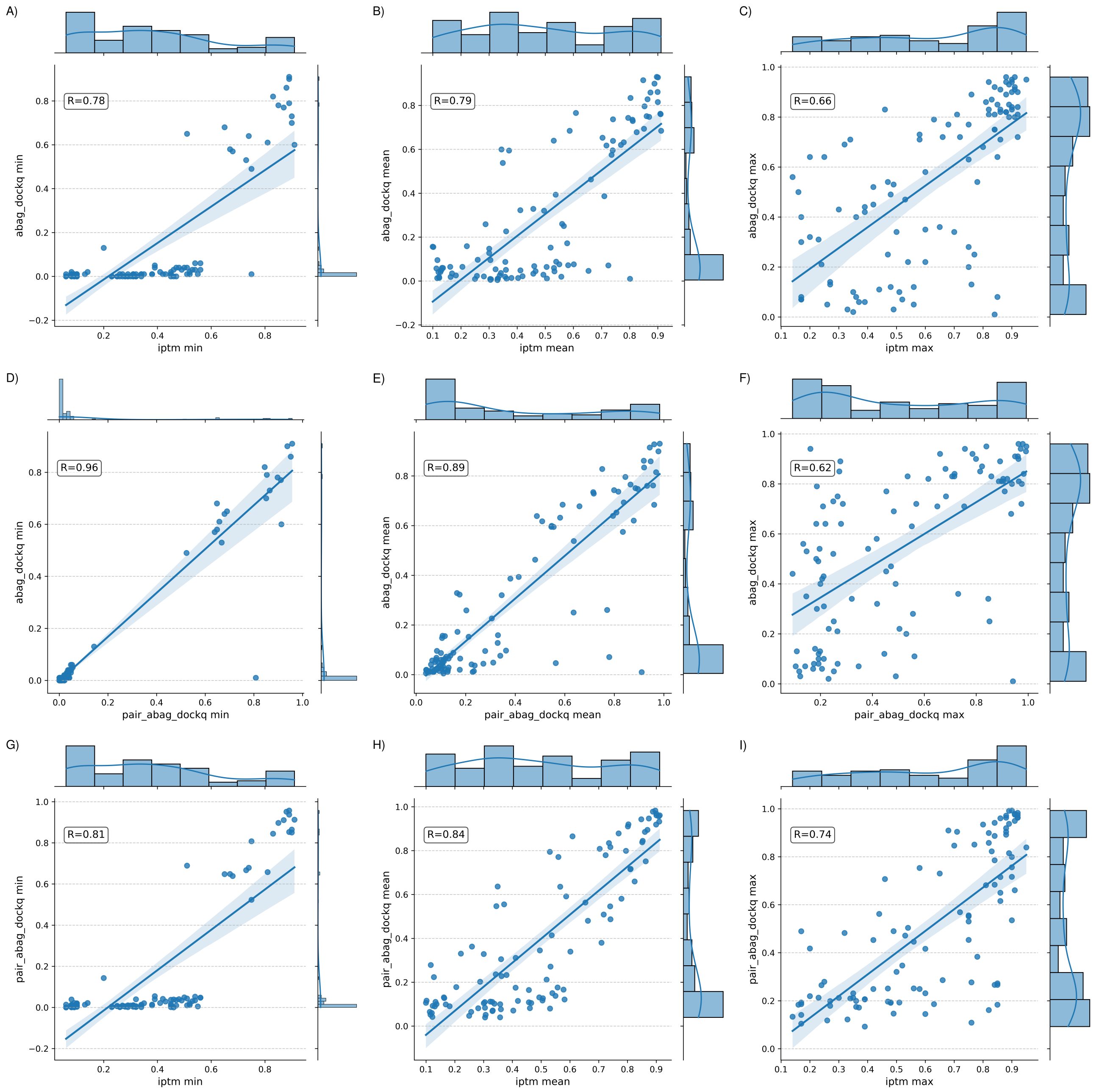

Fig. S13. Comparison of DockQ, Paired DockQ and ipTM for min, mean, and max scores for each target. Overall, all three measures correlate well, particularly when using the min or mean estimates, but Paired DockQ is a better predictor of DockQ than ipTM. One can also note that there is one notable outlier (8us3) that has a high ipTM and Paired DockQ scores but a very low DockQ score, i.e. it is a consistent high-scoring prediction that is wrong, a false positive, no matter what method is used to estimate its quality. The reason 8u3s is wrong is because of its annotation in SabDAB, here the annotated unit corresponds to the assymetric unit, which do not correspond to the the biological assembly.

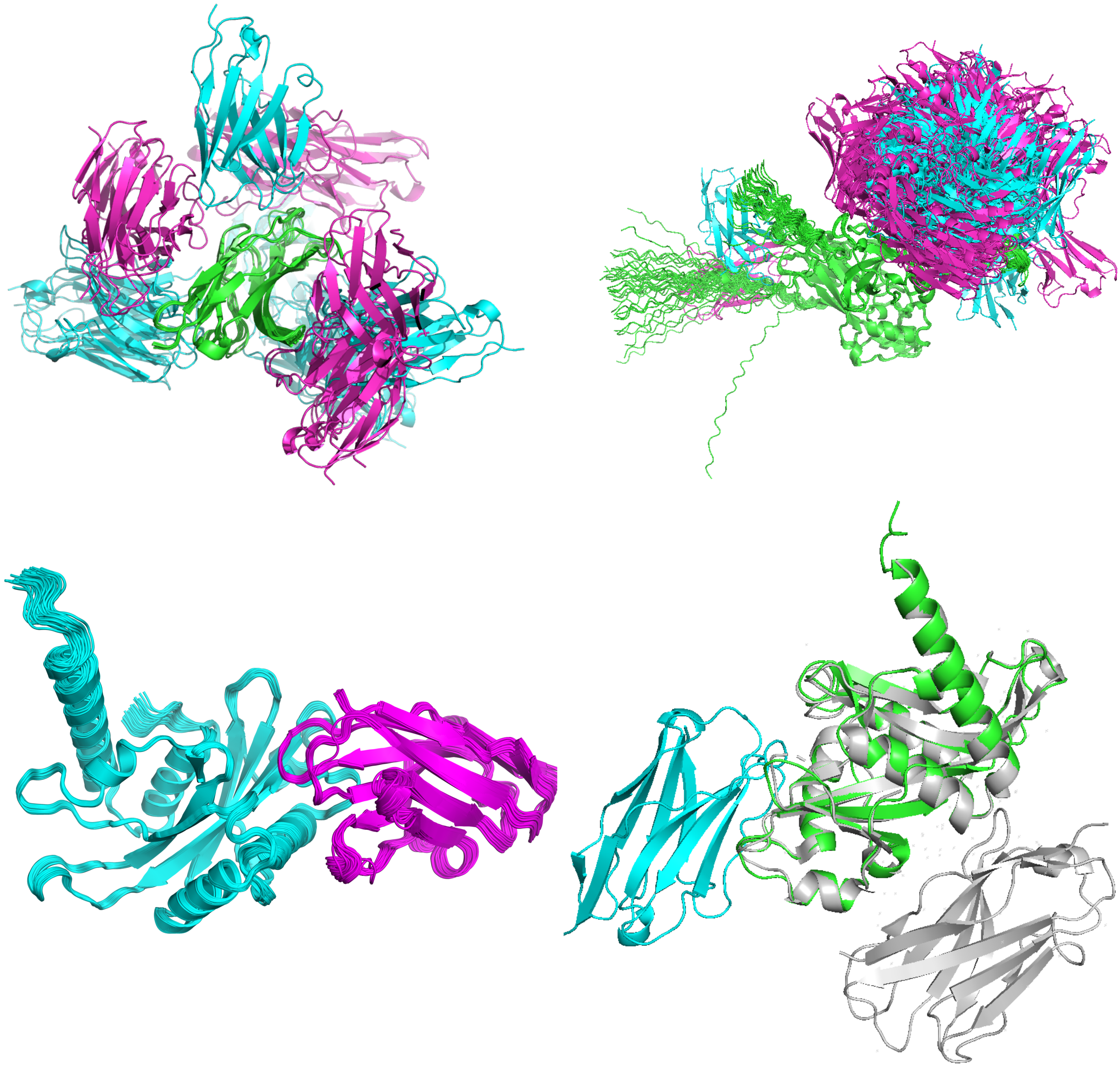

Fig. S14. Example of predictions (all 40 sample 1 models) of predictions using AlphaFold3. All models are superposed on the antigen. A) 8hit (average Paired DockQ= 0.11, ipTM=0.54), B) 8jg5 (average Paired DockQ=0.09, ipTM=0.3) and C) 8U3S (average Paired DockQ= 0.91, ipTM=0.80) D) One prediction of 8U3S against the pdb file (in grey).

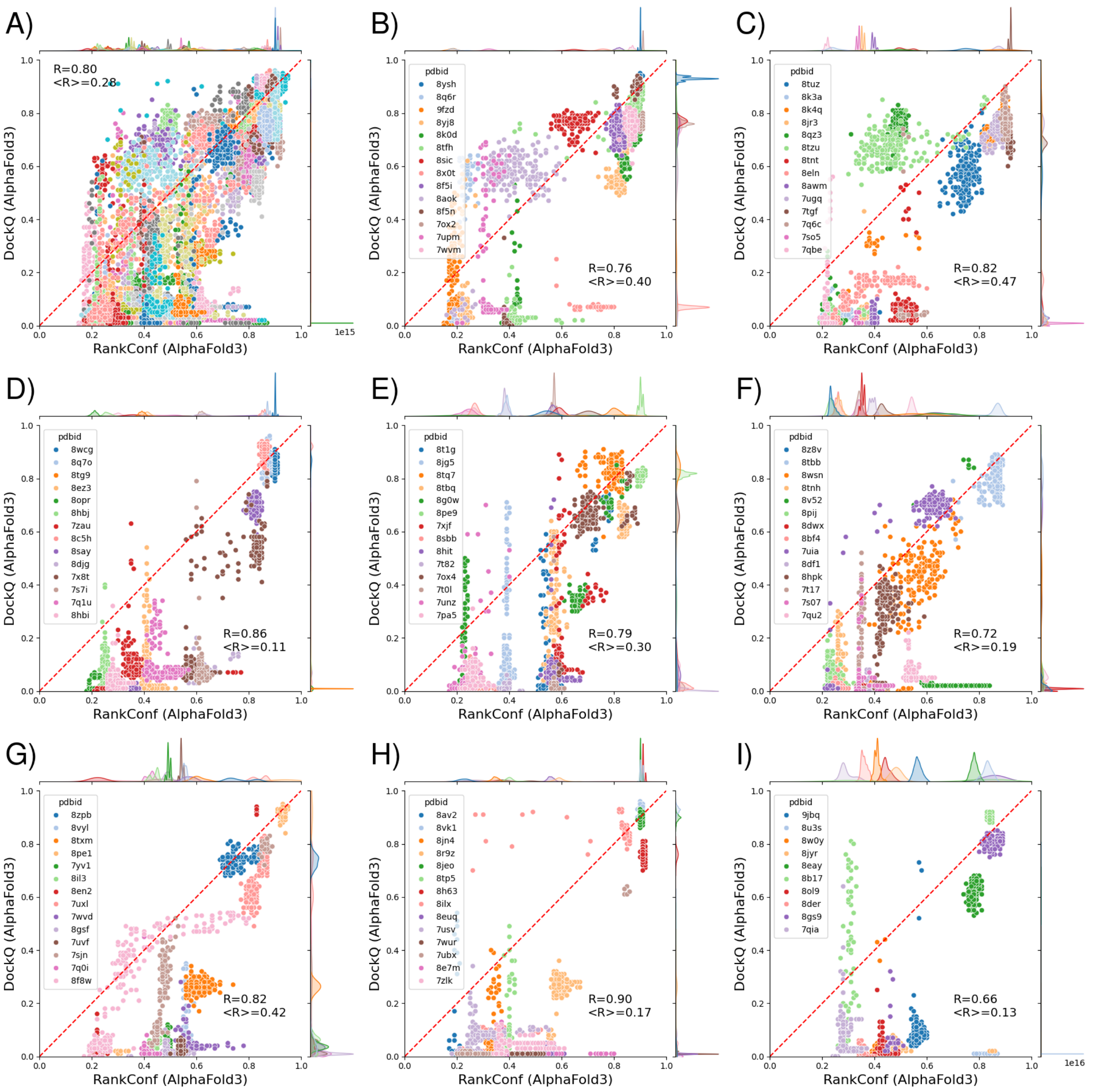

Fig. S15. Scatter plot of Ranking Confidence vs AbAg-DockQ for individual targets. Ten targets per group, each coloured differently. In Figure A all are plotted together.

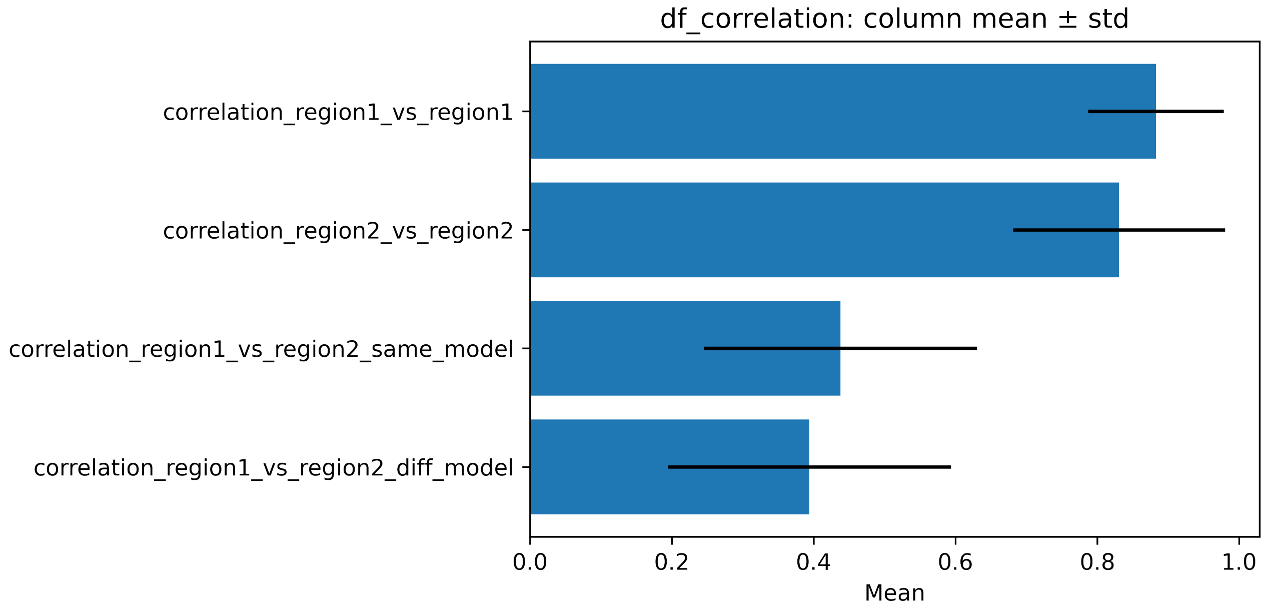

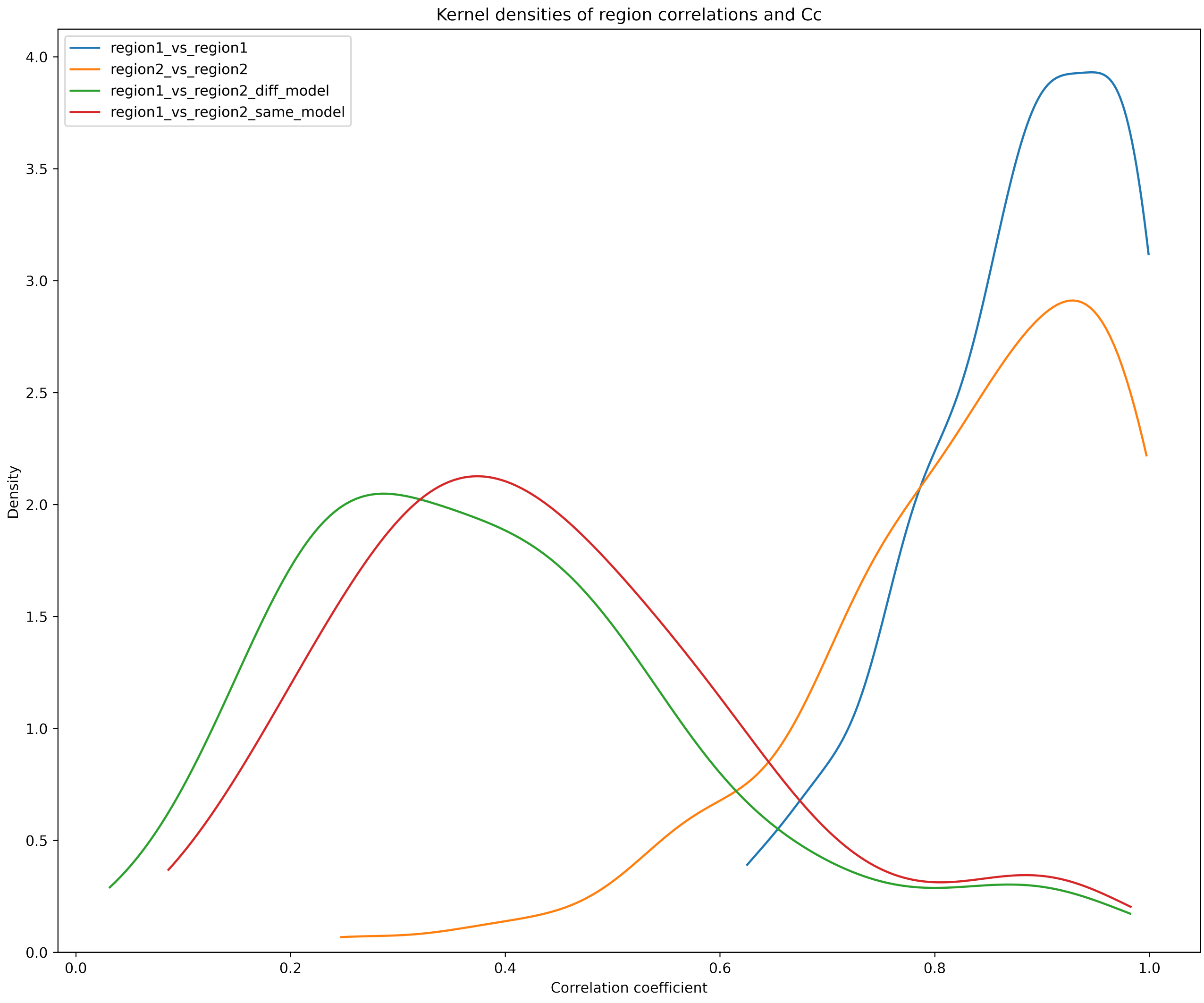

Fig. S16. Top Figure: Correlation between the same and different regions for all models generated for one target. Region 1 is the upper region as shown in Figure 7, i.e. the PAEs when aligned on the antigen, while Region2 relates to the PAEs when aligned on antibody. Bottom Figure: The same distributions in a density plot.

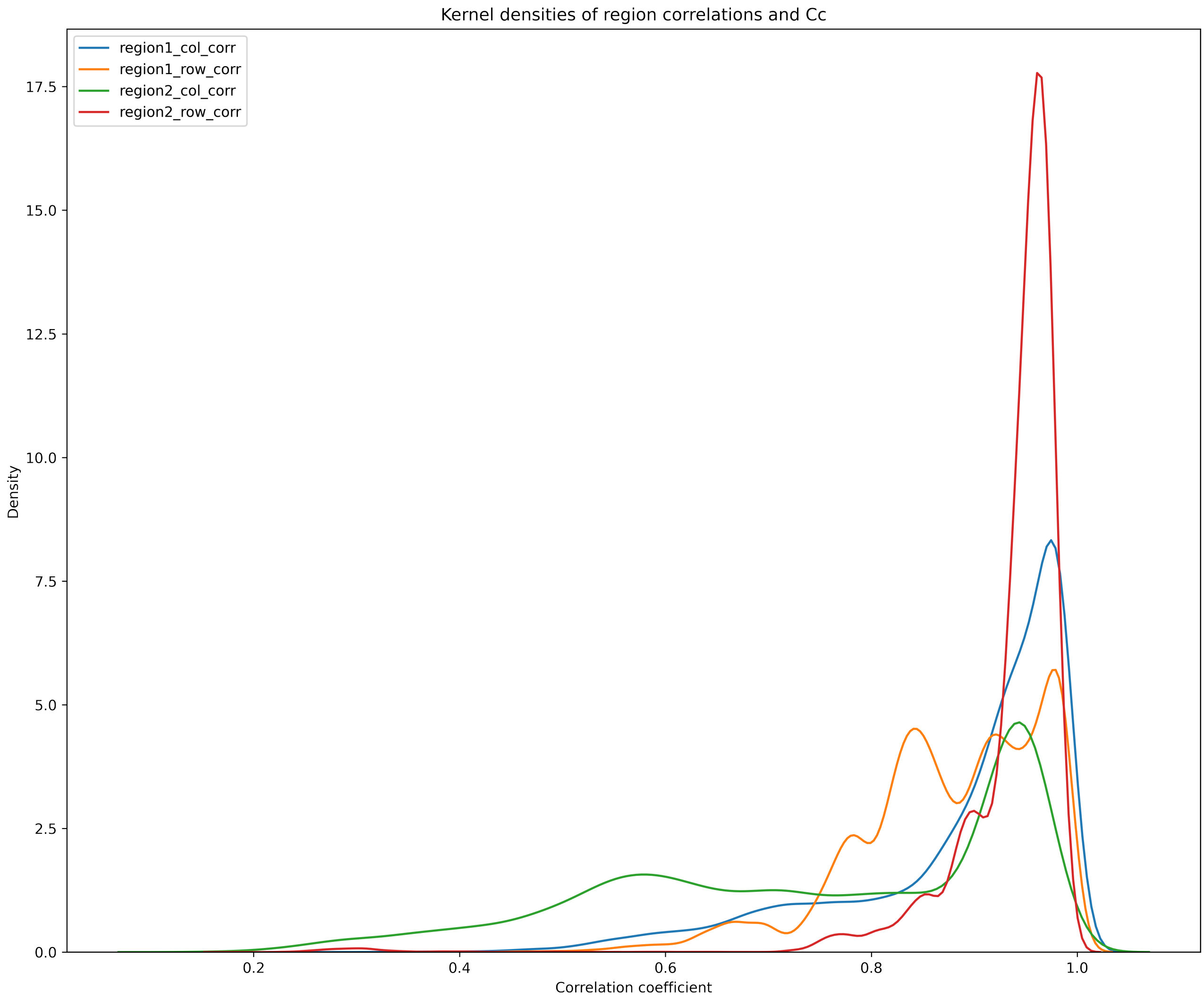

Fig. S17. Correlation within rows/columns in region1 and region2, i.e. how similar are the PAEs when "aligning" on different residues in the antibody (region2) or antigen (region1). The highest correlations are found for region2 row correlation, followed by region1 column correlation, which are the PAEs for residues in the antigen; i.e., there are residues in the antigen that always have higher/lower PAEs than the others.
